# Supplementary material for: The Class I HDAC Inhibitor, MS-275, Prevents Oxaliplatin-Induced Chronic Neuropathy and Potentiates Its Antiproliferative Activity in Mice
Source: Int J Mol Sci. 2021 Dec 22;23(1):98. doi: 10.3390/ijms23010098 (PMC8745279; doi:10.3390/ijms23010098)
Supplement: Supplementary file 1 [file ijms-23-00098-s001.zip › Supplementary Table 1.pdf]

Supplementary Table S1

RESEARCH ARTICLE:

Title: The class I HDAC inhibitor, MS-275, prevents oxali-platin-induced chronic neuropathy and potentiates its antipro-liferative activity in mice

Authors: Sylvain LAMOINE[1†], Mélissa CUMENAL[1†], David A. BARRIERE[1], Vanessa PEREIRA[1], Mathilde FEREYROLLES[1], Laëtitia PRIVAL[1], Julie BARBIER[1], Ludivine BOUDIEU[1], Emilie BRASSET[3], Benjamin BERTIN[3], Yoan RENAUD[3], Elisabeth MIOT-NOIRAULT[4], Marie-Ange CIVIALE[5], David BALAYSSAC[1,6], Youssef AISSOUNI[1], Alain ESCHALIER[1,2] and Jérôme BUSSEROLLES[1*]

FIGURE NUMBER	TEST USED?	n = ?	DEFINED?	REPORTED?	ANOVA MAIN EFFECTS	P VALUE	DEGREE OF FREEDOM & F/t/z/R etc value	SECTION & PARAGRAPH
1B kinetics	Two-way RM ANOVA	9 per group	mice from 2 groups	mean +/- SM	TREATMENT<br>TIME<br>INTERACTION	p < 0.0001<br>p < 0.0001<br>p < 0.0001	F(1.16) = 160.1<br>F(4.64) = 18.72<br>F(4.64) = 38.24	Fig. legend
1C Kinetics	Two-way RM ANOVA	9 per goup	mice from 2 groups	mean +/- SM	TREATMENT<br>TIME<br>INTERACTION	p < 0.0001<br>p = 0.0066<br>p = 0.0193	F(1.16) = 35.21<br>F(4.64) = 3.917<br>F(4.64) = 3.171	Fig. legend
3B Kinetics	Two-way RM ANOVA	6 per goup	mice from 4 groups	mean +/- SM	TREATMENT<br>TIME<br>INTERACTION	p < 0.0001<br>p = 0.0004<br>p = 0.0174	F(3.2) = 38.62<br>F(4.8) = 5.698<br>F(12.8) = 2.232	Fig. legend
3C Kinetics	Two-way RM ANOVA	6 per group	mice from 4 groups	mean +/- SM	TREATMENT<br>TIME<br>INTERACTION	p = 0.0002<br>p < 0.0001<br>p = 0.0035	F(3.2) = 10.38<br>F(4.8) = 14.71<br>F(12.8) = 2.737	Fig. legend
3D kinetics	Two-way RM ANOVA	6 per group	mice from 4 groups	mean +/- SM	SPADIN<br>RILUZOLE<br>INTERACTION	p = 0.0143<br>p < 0.0001<br>p < 0.0001	F(3.2) = 4.453<br>F(4.8) = 7.865<br>F(12.8) = 5.329	Fig. legend
3E	Two-way ANOVA	6 per group	mice from 4 groups	mean +/- SM	OXALIPLATIN<br>MS-275<br>INTERACTION	p < 0.0001<br>p = 0.7926<br>p = 0.0805	F(1.2) = 45.39<br>F(1.2) = 0.071<br>F(1.2) = 3.389	Fig. legend
3F	Two-way ANOVA	6 per group	mice from 4 groups	mean +/- SM	OXALIPLATIN<br>MS-275<br>INTERACTION	p = 0.5441<br>p < 0.0001<br>p = 0.6229	F(1.2) = 0.3796<br>F(1.2) = 34.04<br>F(1.2) 2= 0.2488	Fig. legend
3G	Two-way ANOVA	6 per group	mice from 4 groups	mean +/- SM	OXALIPLATIN<br>MS-275<br>INTERACTION	p = 0.5638<br>p = 0.6911<br>p = 0.9272	F(1.2) = 0.344<br>F(1.2) = 0.1624<br>F(1.2) = 0.0086	Fig. legend
3H	Two-way ANOVA	6 per group	mice from 4 groups	mean +/- SM	OXALIPLATIN<br>MS-275<br>INTERACTION	p = 0.2037<br>p = 0.6351<br>p = 0.0443	F(1.2) = 1.727<br>F(1.2) = 0.2322<br>F(1.2) = 4.608	Fig. legend
4B D0	Two-way ANOVA	6-15 per group	mice from 4 groups	mean +/- SM	OXALIPLATIN<br>MS-275<br>INTERACTION	p = 0.8212<br>p = 0.3707<br>p = 0.7309	F(1.41) = 0.0518<br>F(1.41) = 0.8193<br>F(1.41) = 0.1199	Fig. legend
4B D3	Two-way ANOVA	6-15 per group	mice from 4 groups	mean +/- SM	OXALIPLATIN<br>MS-275<br>INTERACTION	p = 0.3636<br>p = 0.4121<br>p = 0.4	F(1.41) = 0.8442<br>F(1.41) = 0.6866<br>F(1.41) = 0.7232	Fig. legend
4B D7	Two-way ANOVA	6-15 per group	mice from 4 groups	mean +/- SM	OXALIPLATIN<br>MS-275<br>INTERACTION	p = 0.0973<br>p = 0.004<br>p = 0.0796	F(1.41) = 2.879<br>F(1.41) = 9.301<br>F(1.41) = 3.231	Fig. legend

4B D10	Two-way ANOVA	6-15 per group	mice from 4 groups	mean +/- SM	OXALIPLATIN<br>MS-275<br>INTERACTION	p = 0.0953<br>p = 0.0011<br>p = 0.1298	F(1.41) = 2.915<br>F(1.41) = 12.41<br>F(1.41) = 23.9	Fig. legend
Table 1	Kruskal-Wallis ANOVA	6-15 per group	mice from 4 groups	mean surviva l		p = 0.0018		Fig. legend
5A	Two-way ANOVA	3, 3	T84 cells from 2 groups	mean +/- SM	TREATMENT<br>DOSE<br>INTERACTION	p < 0.0001<br>p < 0.0001<br>p < 0.0001	F(2.6) = 327.8<br>F(7.42) = 29.45<br>F(14.42) = 4.762	Fig. legend
5B	Two-way ANOVA	3, 3	HT29 cells from 2 groups	mean +/- SM	TREATMENT<br>DOSE<br>INTERACTION	p < 0.0001<br>p < 0.0001<br>p < 0.0001	F(2.6) = 138.0<br>F(7.42) = 158.4<br>F(14.42) =15.2	Fig. legend
5C	Two-way ANOVA	3, 3, 3, 3	T84 cells from 4 groups	mean +/- SM	OXALIPLATIN<br>MS-275<br>INTERACTION	p < 0.0001<br>p = 0.9492<br>p = 0.1667	F(1.8) = 263.8<br>F(1.8) = 0.0043<br>F(1.8) = 2.314	Fig. legend
5D	Two-way ANOVA	3, 3, 3, 3	HT29 cells from 4 groups	mean +/- SM	OXALIPLATIN<br>MS-275<br>INTERACTION	p < 0.0001<br>p < 0.0001<br>p = 0.0001	F(1.8) = 181.9<br>F(1.8) = 64.65<br>F(1.8) = 50.50	Fig. legend
5E G0/G1	Two-way ANOVA	3, 3, 3, 3	T84 cells from 4 groups	mean +/- SM	OXALIPLATIN<br>MS-275<br>INTERACTION	p < 0.0001<br>p = 0.1126<br>p = 0.1714	F(1.8) = 489.1<br>F(1.8) = 3.176<br>F(1.8) = 2.257	Fig. legend
5E S	Two-way ANOVA	3, 3, 3, 3	HT29 cells from 4 groups	mean +/- SM	OXALIPLATIN<br>MS-275<br>INTERACTION	p < 0.0001<br>p = 0.1269<br>p = 0.1634	F(1.8) = 162.2<br>F(1.8) = 2.902<br>F(1.8) = 2.355	Fig. legend
5E G2/M	Two-way ANOVA	3, 3, 3, 3	T84 cells from 4 groups	mean +/- SM	OXALIPLATIN<br>MS-275<br>INTERACTION	p = 0.4514<br>p = 0.3629<br>p = 0.3619	F(1.8) = 0.6266<br>F(1.8) = 0.9309<br>F(1.8) = 0.9348	Fig. legend
5F G0/G1	Two-way ANOVA	3, 3, 3, 3	HT29 cells from 4 groups	mean +/- SM	OXALIPLATIN<br>MS-275<br>INTERACTION	p < 0.0001<br>p < 0.0001<br>p = 0.0192	F(1.8) = 364.5<br>F(1.8) = 66.84<br>F(1.8) = 8.554	Fig. legend
5F S	Two-way ANOVA	3, 3, 3, 3	T84 cells from 4 groups	mean +/- SM	OXALIPLATIN<br>MS-275<br>INTERACTION	p < 0.0001<br>p < 0.0001<br>p = 0.6678	F(1.8) = 75.07<br>F(1.8) = 89.5<br>F(1.8) = 0.1984	Fig. legend
5F G2/M	Two-way ANOVA	3, 3, 3, 3	HT29 cells from 4 groups	mean +/- SM	OXALIPLATIN<br>MS-275<br>INTERACTION	p = 0.0001<br>p = 0.0026<br>p = 0.0112	F(1.8) = 48.52<br>F(1.8) = 18.45<br>F(1.8) = 10.75	Fig. legend
6C	Two-way ANOVA	5, 5, 5, 5	T84 cells from 4 groups	mean +/- SM	OXALIPLATIN<br>MS-275<br>INTERACTION	p = 0.0004<br>p = 0.5484<br>p = 0.9226	F(1.16) = 19.67<br>F(1.16) = 0.3760<br>F(1.16) = 0.009	Fig. legend
6D	Two-way ANOVA	5, 5, 5, 5	HT29 cells from 4 groups	mean +/- SM	OXALIPLATIN<br>MS-275<br>INTERACTION	p = 0.0020<br>p = 0.0002<br>p = 0.3784	F(1.16) = 13.65<br>F(1.16) = 23.34<br>F(1.16) = 0.8207	Fig. legend
6E	Two-way ANOVA	5, 5, 5, 5	T84 cells from 4 groups	mean +/- SM	OXALIPLATIN<br>MS-275<br>INTERACTION	p = 0.0126<br>p = 0.5945<br>p = 0.6008	F(1.16) = 7.892<br>F(1.16) = 0.2950<br>F(1.16) = 0.2849	Fig. legend
6F	Two-way ANOVA	5, 5, 5, 5	HT29 cells from 4 groups	mean +/- SM	OXALIPLATIN<br>MS-275<br>INTERACTION	p < 0.0001<br>p < 0.0001<br>p = 0.0027	F(1.16) = 76.86<br>F(1.16) = 128.1<br>F(1.16) = 12.62	Fig legend
Suppl. 1C	Two-way ANOVA	5, 5, 5, 5	T84 cells from 4 groups	mean +/- SM	OXALIPLATIN<br>MS-275<br>INTERACTION	p = 0.0003<br>p = 0.0002<br>p = 0.6639	F(1.16) = 20.53<br>F(1.16) = 23.38<br>F(1.16) = 0.1959	Fig. legend

Suppl. 1D	Two-way ANOVA	4, 4, 4, 4	HT29 cells from 4 groups	mean +/- SM	OXALIPLATIN<br>MS-275<br>INTERACTION	$p = 0.2046$<br>$p = 0.0077$<br>$p = 0.8588$	$F(1.12) = 1.799$<br>$F(1.12) = 10.2$<br>$F(1.12) = 0.033$	Fig legend
Suppl. 1E	Two-way ANOVA	5, 5, 5, 5	T84 cells from 4 groups	mean +/- SM	OXALIPLATIN<br>MS-275<br>INTERACTION	$p = 0.0004$<br>$p < 0.0001$<br>$p = 0.3297$	$F(1.16) = 19.43$<br>$F(1.16) = 44.31$<br>$F(1.16) = 1.011$	Fig. legend
Suppl. 1F	Two-way ANOVA	5, 5, 5, 5	HT29 cells from 4 groups	mean +/- SM	OXALIPLATIN<br>MS-275<br>INTERACTION	$p < 0.0001$<br>$p < 0.0001$<br>$p = 0.0027$	$F(1.16) = 76.86$<br>$F(1.16) = 128.1$<br>$F(1.16) = 12.62$	Fig legend
