# Supplementary material for: The Class I HDAC Inhibitor, MS-275, Prevents Oxaliplatin-Induced Chronic Neuropathy and Potentiates Its Antiproliferative Activity in Mice
Source: Int J Mol Sci. 2021 Dec 22;23(1):98. doi: 10.3390/ijms23010098 (PMC8745279; doi:10.3390/ijms23010098)
Supplement: Supplementary file 1 [file ijms-23-00098-s001.zip › Table 1.pdf]

Table 1 : Effect of oxaliplatin and MS-275 on mean and median survival

	**Vehicle**	**Oxaliplatin**	**MS-275**	**Oxaliplatin MS-275**
**Mean survival (days)**	16.85 +/- 2.76	25.23 +/- 11.37	30.50 +/- 10.71 *	34.13 +/- 8.79 **
**Median Survival (days)**	17	19	30.5	36
