# Supplementary figures and images for: The Class I HDAC Inhibitor, MS-275, Prevents Oxaliplatin-Induced Chronic Neuropathy and Potentiates Its Antiproliferative Activity in Mice
Source: Int J Mol Sci. 2021 Dec 22;23(1):98. doi: 10.3390/ijms23010098 (PMC8745279; doi:10.3390/ijms23010098)

## Slide 1
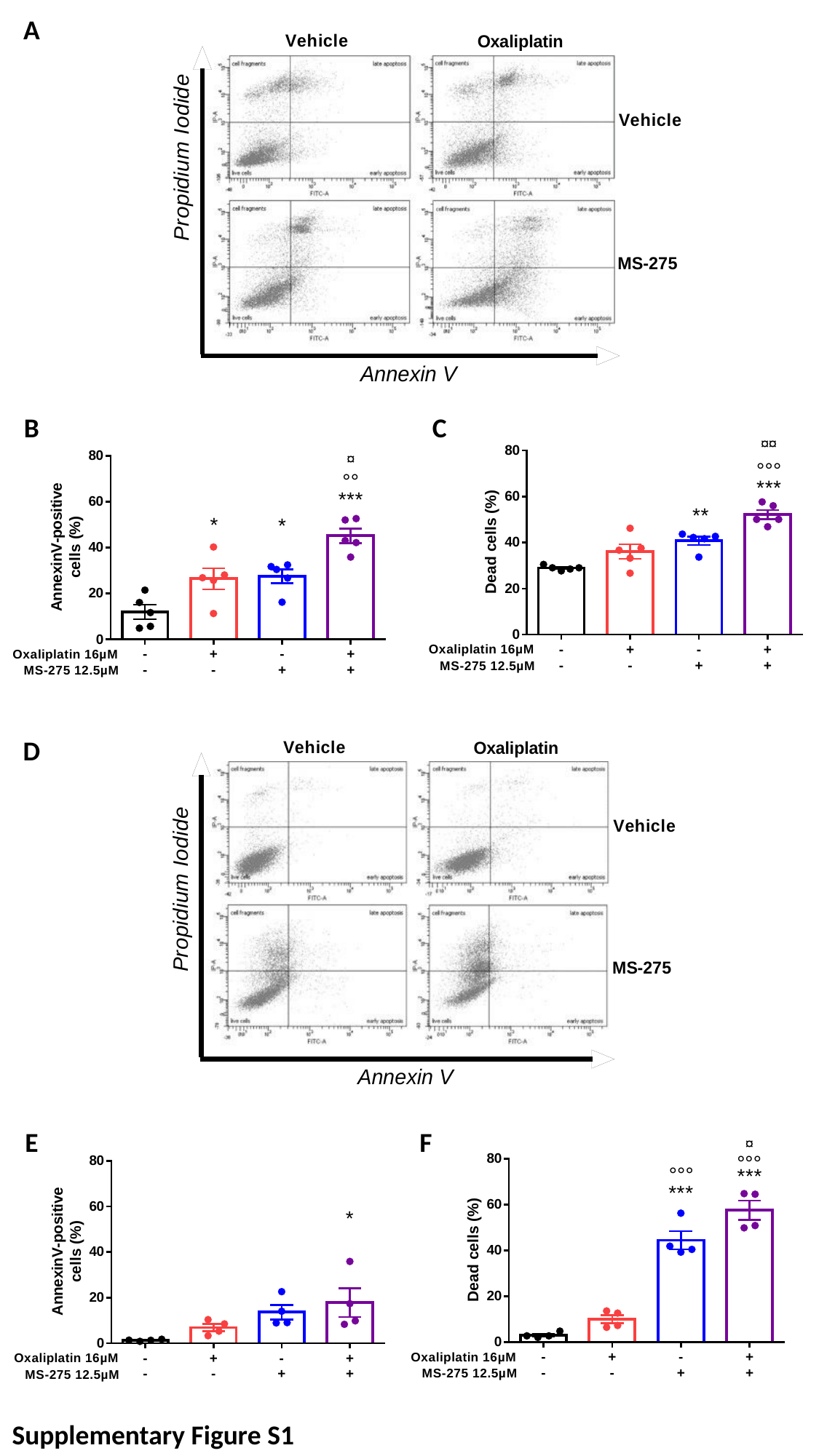

A
B
C
D
E
F
Supplementary Figure S1

Supplement: Supplementary file 1 [file ijms-23-00098-s001.zip › Supplementary Figure S1 revision.pptx]
